# Advances in Dental Materials “at a Glance.”

**DOI:** 10.3390/ma14071750

**Published:** 2021-04-02

**Authors:** Tonino Traini

**Affiliations:** Innovative Technologies in Medicine & Dentistry Department, University “G. D’annunzio” of Chieti-Pescara, Via dei Vestini 31, 66100 Chieti, Italy; tonino.traini@unich.it

Dental materials play a fundamental role in the rehabilitation of tooth structures and regeneration of oral tissues. In the last years, technological development played a key role in the field of dental materials, introducing the engineered materials. These materials exhibiting a “smart behavior“ generated a new era that helps the dental materials to respond to stimuli by altering one or more of their properties without compromising the protection of tooth and oral tissues [1,2]. Likewise, the materials intended for prosthetic restorations were provided with an innovative structure on micro-nano-scales. Similar structural characteristics belong to materials like Zirconia, lithium disilicate or the Zirconia-Lithium-silicate. Some argue that in dental materials it is toughness that is required, not strength or hardness. In this respect, considering also the low adhesive values in cementation, and despite the wide clinical use, zirconia seems to be inadequate. So where are we going? all the more so, the zirconia, with its characteristics of chemical inertia, fails to have an adequate etchable surface when compared with the values obtained with glass ceramics. To overcome these limitations, primers containing 10-methacryloyloxydecyl dihydrogen phosphate (10 MDP) have been developed and introduced in dentistry whose clinical effectiveness remains to be confirmed. To address this issue, Valente et al. [3] have evaluated, in an original article, the resistance values of adhesive cementation of zirconia discs prepared for the clinical use thickness, with different resin-cement added or not with the 10 MDP. The 10-MDP was effective to improve the bond strength between resin-cement and zirconia. Clinically, this new generation of dental materials needs to be validated to enable prediction of long-term results. In this regard, a valuable study was performed by Brandt et al. [4], which reported a 5-year cumulative survival rate of 94.22% obtained on a total of 1058 full-coverage crowns made of IPS e.max Press, IPS e.max CAD, IPS e.max Ceram or IPS e.max ZirPress.

The conventional dental materials have been acquisitive for long, which means they were developed elsewhere for different purposes, and following the principles of ‘adopt, adapt, improve’, sometimes with idiosyncratic and personal recipes of individual practitioners, have been introduced in clinical practice. The evolution and exponential diffusion of digital technologies allowed the development of special materials to be used for CAD/CAM systems. These materials with a digital vocation are more “sophisticated” than those used in analogue techniques. Usually, they are available in partially crystallized form to be milled, after that they must undergo thermal treatment to reach the final crystallization at a well-defined temperature for an exact time, as indicated by manufacturers. Crystallization is an important step performed in the laboratory by dental technician in an oven. Regarding this aspect, an interesting contribution to the use of innovative systems to control and improve the oven’s calibration methods is provided by Duma et al. [5], which focused on employing of optical coherence tomography (OCT) to evaluate the surface structure differences introduced by temperature variations in the crystallized or sintered ceramics to be used in dental laboratory.

With the help of nanotechnology [6], several next-generation materials have emerged on the dental market. This innovative approach, as applied to dentistry, promotes the interaction of organic/inorganic constituents at molecular-level, allowing the incorporation of the material in the living tooth structure complex or favoring implantation and replacement of biomaterials. This arrangement allows for a myriad of possible therapeutic applications [6]. Grazziotin-Soares et al. [7] using an experimental niobium phosphate bioactive glass, as intracanal medication, for 15 days, demonstrate an increase of the dentin microhardness contrary to the calcium hydroxide, which produces a weakening effect in dentin.

Thanks to the progress and materials development, additional revolutionary properties were introduced.

The bioactive property, defined as “the effect eliciting a response from living tissue”, contributes to the formation of a new living compatible system. [8,9].

The bioinductive property is defined as “the capability of a material for inducing a response in a biologic system” [10].

The Biomimetics term was firstly used by the engineer and physicist Schmitt OH, in 1957, to describe a biological approach to engineering [11]. This is a property which define “a material that mimics natural substances for function and appearance” [12].

Most of the reported properties appear to be fundamentals for biomaterials intended for guided tissue regeneration (GTR). De Tullio et al. [13], in a histological human study, reported evidence of additive synergistic action in the case of use of a mix of multiple substitute bone materials. Placed together, they showed associated bioactivity and bioinductivity results. Verardi et al. [14] showed that the combination of nanohydroxyapatite powder and polylactic acid/polyglycolic acid copolymer, used as bone replacement graft in intrabony periodontal defects, may give significant improvements of periodontal parameters at 12-months of follow-up. De Oliveira Rosso et al. [15] in a review evaluated the effects of the photobiomodulation (PBMT) together with bovine bone as a scaffold, in regenerative dentistry. The results showed a potential to improve the bone reconstructive process using PBMT.

The starting stage of any oral reconstruction starts with a diagnostic wax-up and proceeds with a formulation of treatment plan, finally selecting the best-performing materials able to satisfy the clinical need. The minimally invasive dentistry frequently adopts all-ceramic restorations bonded to tooth. This procedure requires a very precise operational protocol that starts from the preparation of the teeth and follows with treatment of the prepared surfaces through immediate dentin sealing (IDS). They end with the cementing of the restorations. Sinjari et al. [16,17], in two consecutive original articles, reported the efficacy of the IDS procedure and documented the penetration of the impression material inside the dentinal tubules strongly supporting the use of the IDS procedure for adhesive restorations.

This special issue comprises original research articles and literature reviews offering solutions and points of view to some current problems in dentistry, providing wide-ranging knowledge about the evidence and current trends in dental materials for restorative and regenerative use; however, future materials will probably go far beyond. Hopefully, readers of this special issue will gain critical understanding of the progress in dental restorations and rehabilitation.

## Data Availability

Not applicable.
